# Femoral head vascular status in early-stage Legg–Calvé–Perthes disease assessed by contrast-enhanced magnetic resonance imaging: comparison with the contralateral side

**DOI:** 10.1186/s13023-025-04074-8

**Published:** 2025-10-22

**Authors:** Shiyu Huang, Xiang Cheng, Sijie Gao, Xinyan Huang, Yinxin Liu, Chuan Feng, Yuxi Su

**Affiliations:** 1 Radiology Department, Children’s Hospital of Chongqing Medical University, Chongqing Key Laboratory of Pediatrics, Ministry of Education Key Laboratory of Child Development and Disorders, National Clinical Research Center for Child Health and Disorders, China International Science and Technology Cooperation Base of Child Development and Critical Disorders, 136# Zhongshan 2road Yuzhong District, Chongqing, 400014 China; 2 Orthopedics Department, Children’s Hospital of Chongqing Medical University, Chongqing Key Laboratory of Pediatrics, Ministry of Education Key Laboratory of Child Development and Disorders, National Clinical Research Center for Child Health and Disorders, China International Science and Technology Cooperation Base of Child Development and Critical Disorders, 136# Zhongshan 2road Yuzhong District, Chongqing, 400014 China

**Keywords:** Legg–Calvé–Perthes disease (LCPD), Femoral head (FH), Cartilaginous vessels, Gadolinium-enhanced magnetic resonance imaging (MRI)

## Abstract

**Background:**

Legg–Calvé–Perthes disease (LCPD) is characterized by avascular necrosis of the femoral head (FH) in children. FH blood supply restoration is important for understanding LCPD’s pathophysiology. We used gadolinium-enhanced magnetic resonance imaging (MRI) to clarify the early-stage FH vascular status in patients with stage I Waldenström LCPD.

**Methods:**

This retrospective study included 23 patients diagnosed with unilateral LCPD using gadolinium-enhanced MRI between January 2017 and September 2024. The vascular evaluation of ossification centers was categorized into visible and invisible levels. Additionally, the axial FH cartilage was classified into medial, lateral, anterior, and posterior parts. Compared with the contralateral normal side, each part’s vascularity on the lesion side was categorized into reduced, comparable, and increased grades. Proportions of grades across parts were compared using Fisher’s exact test with Bonferroni correction. Blood vessel thickness was also assessed.

**Results:**

On the affected side, the FH vascular distribution was mainly concentrated within the cartilage, with the ossification center vessels observed in only seven cases. The proportions of patients with increased cartilaginous vessels in the medial, lateral, anterior, and posterior parts were 65.2%, 78.2%, 8.6%, and 26.0%, respectively. Statistically significant discrepancies were observed in the medial and lateral parts compared to the posterior and anterior parts. Thickened cartilaginous vessels were present in the lateral part of 78.2% of patients. The ratio was 60.8%, 17.3% and 34.7% in the medial, anterior, and posterior parts, respectively. Due to the prolonged LCPD course and absence of outcome data, we did not investigate the relationship between FH vascular status in the early stage and clinical outcomes of LCPD.

**Conclusion:**

In patients with stage I Waldenström LCPD, the affected FH exhibits increased and thickened cartilaginous vessels, suggesting more pronounced vascular remodeling compared to the ossification center. The lateral and medial parts exhibited the most obvious cartilaginous vascular manifestation.

**Level of evidence:**

IV.

## Introduction

Legg–Calvé–Perthes disease (LCPD) is an ischemic necrosis disease of the femoral head (FH) in children, which typically manifests with unilateral or bilateral lesions of the FH. Its incidence among children aged < 15 years ranges between 0.2 and 19.1 per 100,000 [1]. Although the LCPD’s etiology remains unclear, its potential causative factors include interrupted FH blood supply, environment, metabolism, genetics, and stress [2]. Many researchers believe that disruption in the arterial supply to the FH is a key causative factor. In animal models (piglet and rat), interrupted blood supply to the FH induces histopathological and radiographic changes similar to those of LCPD [3, 4]. Experimental studies have demonstrated the ischemic process of LCPD and showed FH changes consistent with avascular necrosis [5, 6]. Therefore, restoration and reconstruction of the FH blood supply play a crucial role in LCPD development and prognosis.

Atsumi et al. [7] conducted super-selective angiography of the hip joint and observed numerous small arteries growing into the ischemic area at different stages of the disease. However, angiography has rarely been used in clinical studies to evaluate FH blood supply in patients with LCPD because of its invasive nature. Magnetic resonance (MR) imaging (MRI) is commonly used in clinical practice to assess changes in the FH blood supply. Perfusion MRI and dynamic gadolinium-enhanced subtraction MRI (DGS-MRI) studies can detect perfusion or enhancement changes in the FH and assess blood supply reconstruction in patients with LCPD [8, 9]. An increase in perfusion or enhancement in the FH in the early stages of LCPD, particularly in the lateral pillar, is significantly correlated with prognosis [10, 11]. Some scholars have also observed a significant correlation between an elevated metaphyseal apparent diffusion coefficient ratio and the absence of an enhanced lateral pillar on DGS-MRI [12]. However, these MRI examinations do not directly reveal the FH blood vessels. In children with open epiphyseal plates, the blood vessels supplying the FH ossification center must pass through the cartilage before entering the ossification center. Therefore, previous studies that focused solely on the ossification center were incomplete. We previously confirmed that a contrast-enhanced MRI of the hip completed within 7 min after contrast injection could display cartilaginous and osseous blood vessels of the FH [13]. Using this method, we aimed to clarify the early-stage vascular status of LCPD by retrospectively studying gadolinium-enhanced MRI results in patients diagnosed with stage I Waldenström LCPD.

## Materials and methods

### Patients

**Fig. 1:**
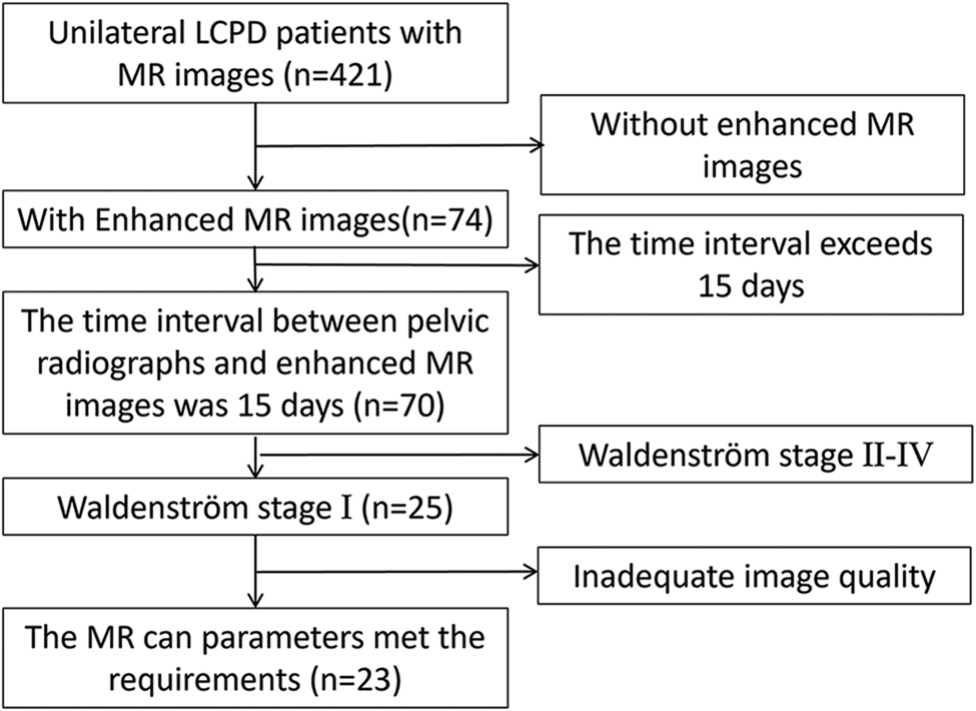
Flowchart of the enrolled patients. MR = magnetic resonance, LCPD = Legg–Calvé–Perthes disease

We retrospectively analyzed the imaging data of patients with unilateral LCPD treated at our hospital between January 2017 and September 2024. Patients with stage I Waldenström LCPD were screened using pelvic radiography. They also underwent pelvic radiography follow-up for over half a year, which confirmed unilateral LCPD. Both pelvic radiographs and enhanced MR images were analyzed. Ultimately, 23 patients were included in this study (Fig. 1). This study was approved by our hospital’s Ethics Committee (No. 2023 − 425), and all patients’ guardians signed a consent form for this study’s publication.

### Pelvic radiographs

Pelvic radiographs were obtained from patients within 15 days pre- or post-MRI session. If multiple pelvic radiographs were obtained during this period, the radiograph with the shortest interval from the time of MR examination was used. The images were examined by two pediatric radiologists with over 10 years of experience. Specifically, the Waldenström stage of the patients was determined based on pelvic radiographs by the two radiologists [12]. Patients who were in Waldenström stage I at the time of the screening were included. Any disagreements regarding staging were resolved through consensus.

### MRI protocol

Among the 23 patients, 10 underwent MRI with 1.5 T scanners (EXCITE, GE Healthcare, Milwaukee, WI, USA), while 13 were scanned using 3.0 T scanners (Achieva 3T, Philips Healthcare, Netherlands [*n* = 10]; Discovery MR750, GE Healthcare, Milwaukee, WI, USA [*n* = 3]). All patients underwent routine MRI, including axial T1-weighted image (T1WI), axial fat-suppressed T2-weighted image (T2WI), coronal T1WI, and coronal fat-suppressed T2WI sequences. A gadolinium contrast agent (0.2 mmol/Kg) was injected after the conventional scan completion, followed by contrast-enhanced scanning. This contrast-enhanced scanning in the axial position was completed within 7 min after contrast injection. The axial voxel and slice thickness did not exceed 1.2 × 1.2 mm and 4 mm, respectively.

### Analysis of MR images

**Fig. 2:**
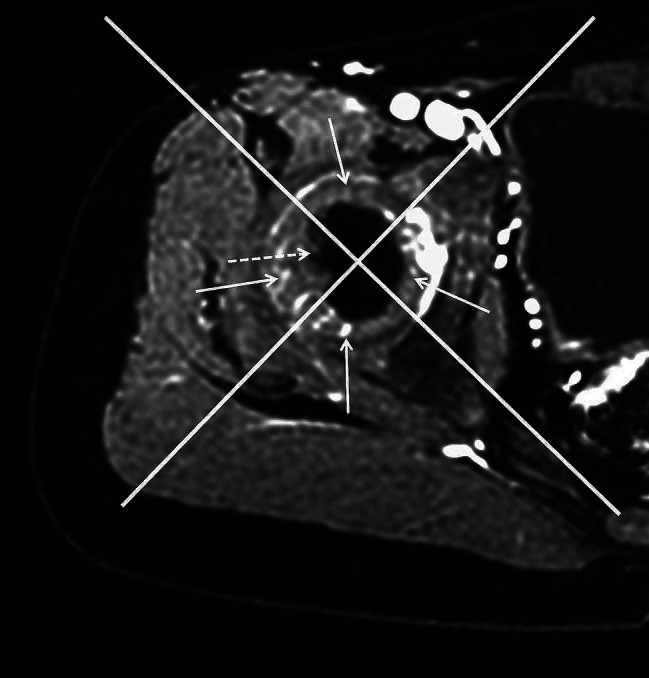
Enhanced MR image of the normal femoral head in a 4-year-old boy. The FH is categorized into four parts (medial, lateral, anterior, and posterior) by two perpendicular straight lines passing through the center of the FH. The 90° area directly in front is defined as the anterior part. The ossification center (dotted arrow) and cartilaginous (arrow) vessels are shown. MR = magnetic resonance, FH = femoral head

A routine MR scan was performed to confirm the T1WI and T2WI signal changes in the FH on the sides of the patients with LCPD. FH vascularity was analyzed using MR axial enhancement by two pediatric radiologists with over 10 years of experience. The vascular evaluation of ossification centers was divided into visible and invisible levels. We connected the linea alba to the coccyx midpoint and established an orthogonal coordinate system with a perpendicular line on the axial image showing the maximum area of the FH. The coordinate system’s origin was moved to the FH center, and subsequently rotated 45° to divide the FH cartilage into the following four parts: anterior, lateral, medial, and posterior (Fig. 2). Each continuous linear or punctate enhancement on the cartilage is counted as one vessel. The number of each part’s cartilaginous vessel was counted at the axial sections on the affected side. Compared with the normal side, the result was categorized into the following three grades: reduced (the cartilaginous vessel represented < 50% of the normal side), increased (the cartilaginous vessel comprised > 50% of the normal side), and comparable (between the two grades). The proportions of each grade in each part were calculated. Furthermore, the width of each cartilaginous vessel on the affected side was measured. The cartilaginous vessel was defined as thickened if its width exceeded the maximum width of the same-region cartilage vessels on the normal side.

### Intra- and inter-observer variability

Intra-observer variability of the cartilaginous vascular number grades was evaluated by a pediatric radiologist, who conducted two blinded assessments at intervals exceeding 2 months between them. The second pediatric radiologist independently assessed inter-observer variability.

### Statistical analysis

Statistical analyses were performed using IBM SPSS Statistics for Windows, version 27.0 (IBM Corp., Armonk, NY, USA). Fisher’s exact test was used to compare the proportions of grades between each part. Bonferroni correction was conducted to correct the bias of multiple testing. Intra- and inter-observer reproducibility were determined using the intraclass correlation coefficient (ICC). Statistical significance was set at *p* < 0.05.

## Results

Twenty-three patients (18 males and 5 females) with LCPD were included in this study. Their mean age was 5.29 ± 1.76 (range, 3.0–9.0) years. All patients had unilateral hip LCPD, including 10 and 13 with left and right hip lesions, respectively (Table 1).

**Table 1:** Demographic data of LCPD cases and the grade of femoral head cartilaginous vascularization

Patient	Age (Years)	Sex	Affected side	Medial part			Lateral part			Anterior part			Posterior part		
				*R*	C	I	*R*	C	I	*R*	C	I	*R*	C	I
1	7	M	Right			+			+		+			+	
2	4.3	M	Left			+			+		+			+	
3	5	F	Right			+			+		+			+	
4	3.5	M	Left			+			+		+			+	
5	4	M	Right			+		+			+				+
6	3.8	M	Left			+			+			+		+	
7	4.2	M	Right			+			+		+			+	
8	6.7	M	Left		+				+		+				+
9	8.4	F	Right		+				+		+			+	
10	6.7	M	Right		+			+			+			+	
11	5.8	F	Left		+				+		+			+	
12	3.0	F	Right		+		+				+		+		
13	4.7	M	Right			+			+		+				+
14	5.8	M	Left			+		+			+				+
15	9	M	Left			+			+		+				+
16	3	M	Right			+			+		+			+	
17	6.5	M	Left			+			+		+			+	
18	5.8	M	Right		+			+			+			+	
19	3.8	M	Right			+			+			+		+	
20	3	M	Left			+			+		+		+		
21	4.8	M	Left			+			+		+				+
22	8	M	Right		+				+		+			+	
23	4.9	F	Right		+				+		+			+	
Total				0	8 (34.7%)	15 (65.2%)	1 (4.3%)	4 (17.3%)	18 (78.2%)	0	21 (91.3%)	2 (8.6%)	2 (8.6%)	15 (65.2%)	6 (26.0%)

M = male, F = female, R = reduced, C = comparable, I = increased, LCPD = Legg–Calvé–Perthes disease

All patients showed increased FH density compared to the contralateral side on plain pelvic radiographs. Three patients had no decrease in FH height, while 20 had a decrease in FH height. Subchondral fracture lines were observed in seven patients, and cysts were present at the femur neck in four patients. The medial gap of the hip joint was wider than that of the contralateral side in six patients. However, none of the patients had hip joint dislocation.

On MRI, the ossification center of the FH on the normal side showed homogeneously high and low signal on T1WI and fat-suppressed T2WI, respectively. The ossification center of the affected FH had a heterogeneous signal. T1WI sequences showed a low signal, while fat-suppressed T2WI sequences showed mixed slightly high signals. No significant difference in the signal of the FH cartilage was found on plain scans between the sides.

The cartilaginous vascularity of the FH on the normal side was more pronounced than that of the ossified central vessels on axial gadolinium-enhanced MRI. Additionally, the cartilaginous vessels of the FH on the normal side were predominantly lateral and medial, with fewer vessels on the anterior and posterior sides. The cartilaginous vessels on the anterior side were the least of the four parts (Fig. 2).

**Fig. 3:**
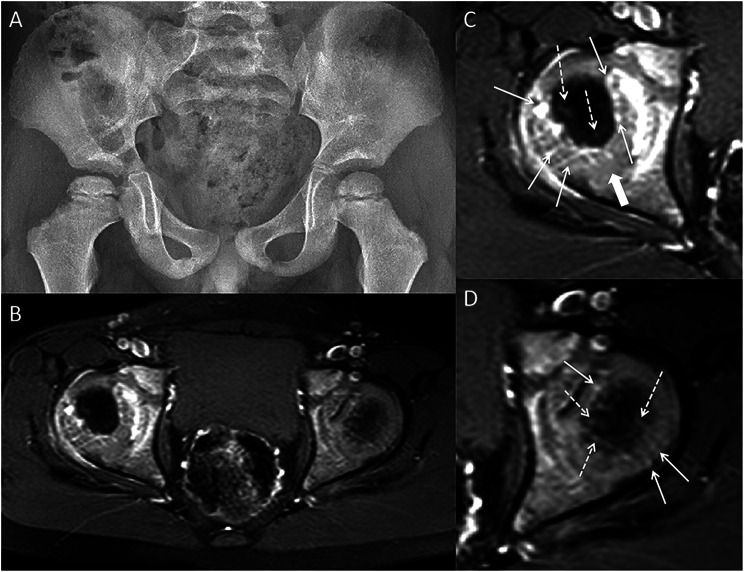
A 4-year-old boy with right LCPD. (**A**) The pelvic radiograph. (**B**) Axial enhanced MR image. (**C**-**D**) The enlarged images of the right and left FH. The ossification center vessels (dotted arrow) are significantly fewer than on the left side. However, the cartilaginous vessels (arrow) in the medial, lateral and posterior parts of the right FH exhibit a more increased grade and are thicker than those on the opposite side. Additionally, the distribution and course of some cartilaginous vessels are similar to those of the normal FH. A zone of relatively sparse vascularity exists in the posterior part (thick arrow). The cartilaginous vessels of the anterior part are the least in the four parts of the bilateral FH. MR = magnetic resonance, FH = femoral head, LCPD = Legg–Calvé–Perthes disease

On the affected FH side, the blood vessels of the ossification center were visible at the edge in seven patients (Fig. 3), while they were invisible in the other patients.

Compared to the unaffected side, the cartilage vessels on the affected FH side were increased in 18 (78.2%) and 15 (65.2%) patients in the lateral and medial parts, respectively (Fig. 3). However, they were increased in only six (26.0%) and two (8.6%) patients in the posterior (primarily on the lateral side—Fig. 3C) and anterior (Table 1) parts, respectively.

Significant differences were observed in the proportion of vessels increasing between the lateral and medial parts compared to the anterior and posterior parts (*p* < 0.008, Bonferroni correction, Table 2).

**Table 2:** Fisher’s exact test of the proportion of vessels increasing across the four parts with bonferroni correction

	Medial part	Lateral part	Anterior part	Posterior part
	*p*	*p*	*p*	*p*
Medial part	-			
Lateral part	≥ 0.008	-		
Anterior part	< 0.008	< 0.008	-	
Posterior part	< 0.008	< 0.008	≥ 0.008	-

p = p value

**Fig. 4:**
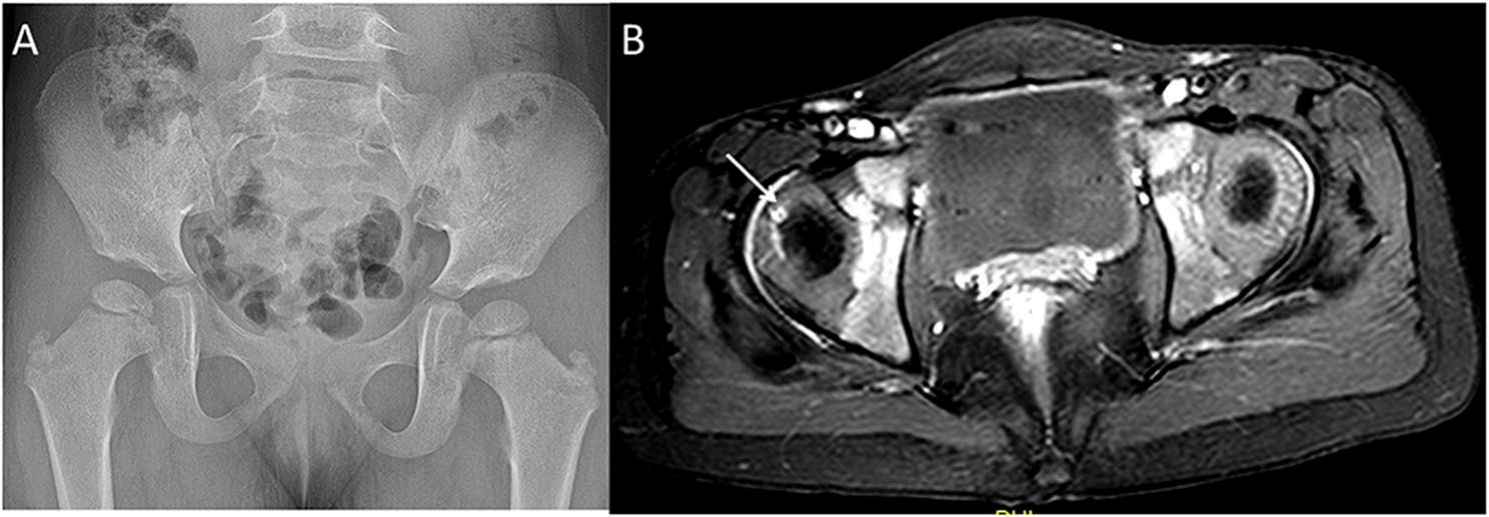
A 3-year-old girl with right LCPD. (**A**) The pelvic radiograph. (**B**) Axial enhanced MR image. The arrow shows the thickened cartilaginous vessels of the right FH; the cartilaginous vessels are fewer than those on the opposite side. MR = magnetic resonance, FH = femoral head, LCPD = Legg–Calvé–Perthes disease

Thickened cartilaginous vessels were found in the lateral and medial parts in 78.2% (18/23) and 60.8% (14/23) of the patients, respectively (Fig. 3). The ratio was 34.7% (8/23) and 17.3% (4/23) in the posterior and anterior parts, respectively. However, the increases and thickening of the cartilaginous vessels were not perfectly matched (Fig. 4).

For intra- and inter-observer agreement of the cartilaginous vascular number grades, the ICC was 0.79 (95% confidence interval [CI]: 0.69–0.85) and 0.74 (95% CI: 0.63–0.82), respectively.

## Discussion

Here, we assessed the differences between the FH blood vessels on the affected and normal sides in patients with LCPD. LCPD is an ischemic form of the FH in children; therefore, restoring the FH blood supply is important for its prognosis. The FH in children has an epiphyseal cartilage, with an ossification center located in the center of this cartilage. Consequently, blood vessels traversing the ossification center must pass through the cartilage before the epiphyseal plate closes, making the reconstruction of cartilage vessels critical in the early stage of LCPD.

In our contrast-enhanced MR images, the patients with stage I Waldenström LCPD had blood vessels in the FH region, suggesting that blood supply restoration begins in the early stages of LCPD. This finding aligns with the results of bone scintigraphy, DGS-MRI, and perfusion MRI for LCPD from previous studies [8, 9, 14]. While these studies showed signal changes in the ossification center of the FH, our images revealed that most of these vessels were present in the cartilage, and a few were present in the bone. This finding suggests that the cartilaginous vessels of the FH are re-established more readily and relatively quickly, while bone vascularization occurs at a slower pace.

The amount of cartilage and cartilaginous vessels varied in our patient group due to age differences. Therefore, we compared the bilateral cartilaginous vessels of the same patients to reduce age-related errors. Based on the distribution of cartilaginous vessels, the lateral and medial parts had most of the vessels. Compared with the contralateral side, the distribution and course of the vessels in these two parts were similar to those on the normal side. A previous animal study has shown that new vessels grew through the ligature of the femoral neck [7, 15]. However, our images suggest that some of the cartilaginous vessels we found originally existed in the FH, as the newly generated vessels should not coincide with the distribution and course of normal vessels.

These two parts align with the anatomical blood supply range of the lateral epiphyseal artery (LEA) and ligamentum teres artery of the FH in healthy children [16]. This is consistent with the findings of previous studies on perfusion MRI and DGS-MRI in patients with LCPD [8, 14]. Atsumi et al. showed blood supply reconstruction from the LEAs was observed in patients with LCPD [7, 15]. In angiographic studies, Atsumi et al. also showed that the ligamentum teres artery plays an important role in blood supply reconstruction in patients with LCPD [17]. Morris et al. reported on the medial and lateral perfusion in patients with LCPD using perfusion MRI [18].

The cartilaginous vessels in the posterior and anterior parts were significantly fewer than those in the lateral and medial parts. Increases in the vessels anterior to the FH are rare. This could be because these two parts are farther away from the main blood supply ranges of the LEA and ligamentum teres artery. The lack of cartilaginous vessels in these two areas during the early stages of LCPD may be due to the reason mentioned above. Previous studies have shown that vessel anastomosis is poorly developed in the anterior part of the femoral neck in some individuals [8, 19]. The posterior part may also have a similar area, resulting in fewer cartilaginous vessels compared to the lateral and medial parts.

Some of the cartilaginous vessels were also shown to be thickened in the FH, particularly in the lateral and medial parts. Johnson et al. [20] studied LCPD in an animal model and found that cartilage vascularization could be a brush-like architecture in a porcine model after 4 weeks of ischemia. We suggested that the thickened cartilaginous vessels we observed could be the brush-like vessels; however, the MRI resolution of our case was insufficient to display these brush-like vessels. Therefore, they appear as thickened cartilaginous vessels on MR images.

We observed that the increased and thickened cartilaginous vessels were present in the early stage of LCPD on MR images, suggesting blood supply restoration to the FH cartilage. Previous studies on LCPD have shown that the lateral pillar height was a key factor affecting prognosis [21, 22]. Perfusion MRI and DGS-MRI also confirmed that blood supply to the lateral column could predict LCPD prognosis [10, 23]. In our study, we found that the cartilaginous vessels were increased and thickened on the lateral and medial parts. We expect that subsequent studies with large sample sizes would determine LCPD prognosis in the early stage. Instead, prognosis can only be evaluated at stage II, as in Herring’s classification [24–28]. Previous morphological studies mainly evaluated the correlation between bony changes and LCPD prognosis [23, 28, 29]. Other studies have demonstrated the correlation between enhancement, perfusion, and diffusion in the FH and prognosis [9, 15, 24, 30]. However, these studies only focused on bony structures or did not distinguish between bone and cartilage. Blood vessels in the ossification center originate from the FH cartilage. Therefore, evaluating the skeletal structure of patients with LCPD alone may not fully reflect the early recovery of blood supply. As presented in Table 1, we observed that the status of cartilaginous vessels in our cases differed from that on the normal side. However, only 30.4% (7/23) of the patients had blood vessels found in the ossification center of the affected FH side. This suggests that the vascular changes could have occurred earlier in the cartilaginous vessels than in the ossification center vessels.

Additionally, thickening of the lateral and medial cartilaginous vessels may lead to more rapid growth of the FH to the sides, resulting in a richer blood supply to the two parts of the FH than normal. This may also explain why the affected FH eventually becomes wider than normal.

In pediatric LCPD, treatment primarily focuses on biomechanical containment through physical therapy, orthotics, or femoral and/or pelvic osteotomies, rather than direct vascular intervention. Therefore, while our findings may enhance the understanding of LCPD’s pathophysiology, they do not advocate new treatment directions.

This study has some limitations. First, the small sample size of 23 patients may affect the internal validity of our results. Therefore, multicenter studies with larger sample sizes are needed to obtain more precise results. Second, our patients were in Waldenström stage I of LCPD based on pelvic radiograph screening, excluding those in the very early stages of LCPD where the FH had no bone changes on pelvic radiographs. Our results may not have included images of LCPD ischemia initiation. Finally, due to the long duration of LCPD and lack of outcome data, we were unable to study the relationship between FH vascular status in the early stage and clinical outcomes of LCPD.

In summary, we observed increased and thickened cartilaginous vessels of FH on enhanced MRI in patients with stage I Waldenström LCPD. The lateral and medial parts of the most affected FH sides exhibited the most obvious manifestation‌, while the cartilaginous vessels in the anterior and posterior parts were poor, particularly in the anterior part. This may serve as an observable indicator of blood vessel reconstruction in patients with early-stage LCPD.

## Data Availability

The data cannot be openly shared due to the need to protect study participant privacy; it can be provided upon reasonable request to the corresponding author.
